# UWB Base Station Deployment Optimization Method Considering NLOS Effects Based on Levy Flight-Improved Particle Swarm Optimizer

**DOI:** 10.3390/s25061785

**Published:** 2025-03-13

**Authors:** Shengliang Wang, Ming Gao, Ling’ai Li, Dong Lv, Yingqi Li

**Affiliations:** 1 State Key Laboratory of Satellite Navigation System and Equipment Technology, Shijiazhuang 050081, China; 2 State Key Laboratory of Precision Geodesy, Innovation Academy for Precision Measurement Science and Technology, CAS, Wuhan 430077, China; gaoming@aircas.ac.cn; 3 School of Vehicle and Transportation Engineering, Taiyuan University of Science and Technology, Taiyuan 030024, China; 202224020116@stu.tyust.edu.cn; 4 College of Mining Engineering, Taiyuan University of Technology, Taiyuan 030024, China; 5 Aerospace Information Research Institute, Chinese Academy of Sciences, Beijing 100094, China; 6 School of Geodesy and Geomatics, Wuhan University, Wuhan 430079, China; lla2019@whu.edu.cn; 7 School of Geographic Information and Tourism, Chuzhou University, Chuzhou 239000, China; lvdong@chzu.edu.cn

**Keywords:** UWB, base station deployment optimization, NLOS, particle swarm optimization, Lévy flight

## Abstract

The ultra-wideband (UWB) base station (BS) deployment pattern seriously affects mobile tag positioning accuracy, but the traditional classical deployment methods, such as rectangular and diamond deployment, cannot take into account the influence of non-line-of-sight (NLOS) occlusion, which leads to a blind area in positioning. In this paper, we propose a new UWB BS deployment optimization method that takes into account the influence of NLOS occlusion, determines the BS deployment range and occlusion by indoor map information, uses the locatable points coverage rate in the whole indoor positioning area as the fitness function, and proposes an improved particle swarm optimization algorithm based on the Levy flight strategy (LPSO) to solve the optimization problem. The simulation experiment results show that the locatable space coverage rate of rectangular and diamond deployment models gradually decreases and the blind positioning area gradually increases with the increase in NLOS occlusion. The locatable space coverage rate of the LPSO-optimized deployment is better than that of the standard PSO-optimized deployment model, while it is 19.0% and 22.6% better than the rectangular deployment and 3.0% and 6.5% better than the diamond deployment when the NLOS values are 3 and 5 for complex occlusion environments, respectively. The experimental results of the underground garage demonstrate that the optimal 13 BS layout scheme, obtained through LPSO, outperforms the 7 BS layout scheme by 34.9% while reducing the horizontal dilution of precision (HDOP) values by 81.7%. Therefore, the proposed UWB BS layout optimization scheme exhibits superior adaptability to large and complex indoor environments, effectively enhances signal coverage and positioning accuracy, and holds significant practical value.

## 1. Introduction

With the development of wireless communication technology, the demand for location-based services (LBS) in people’s daily life and economic production is increasing, and the Global Navigation Satellite System has become very mature and can provide reliable positioning and navigation services with different degrees of precision in outdoor open environments [1]. However, its positioning signals cannot be transmitted directly indoors or underground, and can only be used in open environments [2]. According to statistics, 80% of people’s daily life is spent in indoor space. It is challenging to deliver reliable location services indoors due to the significant GNSS signal dilution caused by structures and other barriers; hence, increasing indoor positioning accuracy has emerged as a key research topic.

Many indoor positioning technologies have advanced quickly in recent years, including Wi-Fi [3], Bluetooth [4,5], Pedestrian Dead Reckoning [6], computer vision [7,8], and UWB [9,10]. Wi-Fi, Bluetooth, and Pedestrian Dead Reckoning have meter-level positioning accuracy, which is insufficient to address the demand for very accurate indoor positioning. Although computer vision can provide positioning accuracy down to the submeter level, its position-solving complexity and energy requirements make it challenging to spread and use [7]. UWB positioning accuracy is superior at the cm level and is utilized extensively in industrial processing, monitoring, tracking, and other applications thanks to its exceptionally high time resolution, huge bandwidth, and low power consumption [11,12]. It is favored by users and regarded as one of the foundational wireless communication technologies in the future. On the other hand, the UWB positioning system is dependent on BSs or beacons for positioning. Mobile tags must be located using the known locations of numerous UWB BSs in the positioning area. The four factors primarily affecting the UWB positioning accuracy are time synchronization or distance measurement error [13], BS site error, non-line-of-sight (NLOS) propagation error [14], and the geometric deployment or configuration of BS [15]. Among them, the time synchronization or ranging error and the BS site error are relatively fixed. The primary element influencing the location accuracy of the UWB tag is the NLOS propagation error. In particular, in an NLOS environment with occlusion, the UWB pulse signal is refracted or reflected to extend the propagation time because the obstruction prevents the signal from passing through directly. This causes a significant range error, which further reduces positioning accuracy. It takes a compensating algorithm to reduce or eliminate the impact of NLOS.

The deployment of the UWB BS has a significant impact on the positioning system’s construction costs, accuracy, and stability. It has been demonstrated that the influence of BS deployment on location accuracy is greater than measurement noise and the fast fading effect [13,16]. Optimizing the BS deployment or configuration in accordance with the varied physical contexts and accuracy requirements is essential for the widespread usage of UWB positioning systems. The magnitude of the dilution of precision (DOP) and the scope of the locatable space represent the impact of the UWB BS deployment or configuration on positioning accuracy in various application situations. Currently, the vast majority of UWB BS deployment for indoor positioning relies primarily on personnel experience, selects traditional ideal formations (such as trilateral, rectangular, rhombus, star, and so on) solely based on scene structure [17], and only uses the DOP value as an analytical or evaluation index to compare several BS deployment methods. In an obstacle-filled environment, the impact of NLOS propagation on UWB positioning accuracy and the cost of BS deployment is ignored, rendering traditional optimization methods inapplicable. Jiang et al. [18] proposed a practical and cost-effective UWB BS deployment scheme for indoor automatic valet parking (AVP). This included deciding on the deployment strategy and deployment parameters and conducting a thorough analysis to adjust and validate the scheme. The scheme took into account the accuracy requirements of AVP in various partitions, such as ramp area, surface fluctuation area, and narrow area, with fewer UWB BSs that ensure stability and economy. It also can meet the localization accuracy requirements of AVP. Zhang et al. [15] explored the effectiveness of UWB motion target tracking methods and BS deployment and discussed the localization performance of linear and nonlinear localization estimators for patterns of rectangular and triangular BS deployment. Wang et al. [19] demonstrated that optimizing UWB BS deployment, with DOP as the optimization target, does not result in the best BS configuration, that DOP should be used as a reference index rather than a decisive index for optimizing UWB BS deployment, and that the optimized BS deployment with the time-difference-of-arrival (TDOA) measurement error as the optimization target results in higher positioning accuracy. Yu et al. [20] investigated the BS deployment problem in cellular network positioning applications, using the indoor 3D localization signal coverable range as a fitness function and an adaptive genetic algorithm with fused simulated annealing for optimization. After optimizing the BS deployment, effective coverage rate increased from 89.77 to 100% and the average positioning error decreased from 2.874 m to 0.983 m, but the study did not consider the effect of NLOS on positioning accuracy and coverage space.

In the actual application of complicated environments, such as shopping malls, underground garage, factories, and other scenes containing both integral rules and irregular spaces, there will be space restrictions imposed by walls and obscurants which will have a significant impact on positioning accuracy. The use of several classical deployments, such as rectangular and diamond deployment, will lead to an insufficient locatable space coverage range with large blind positioning zones and a significant increase in positioning errors. If UWB BS deployment is too dense, although it can improve the positioning accuracy, it will also cause cost increases and be unsuitable for use. Additionally, according to manual experience and classical BS deployment, it is more mechanical and archaic, which cannot guarantee the universality and specificity. As a result, this research suggests an optimization method for the deployment of UWB BSs that takes into consideration the effects of NLOS when seeking to overcome the inadequacies of current BS deployment methods. The goal is to reduce the impact of NLOS signal propagation on UWB positioning, increase the locatable space coverage rate, thus improving positioning accuracy, and at the same time reduce the deployment cost of UWB BSs, providing a flexible and effective optimization method for UWB BS deployment. The key to this process entails modeling the UWB BS deployment problem as an optimization objective or fitness function and solving for the optimal deployment parameters using an improved PSO algorithm based on the Lévy flight strategy (LPSO).

This paper is organized as follows. Section 2 introduces the principle of PSO algorithms based on the improved Levy flight strategy; Section 3 gives the mathematical model and solution steps of UWB BS deployment optimization, taking into account the effect of NLOS occlusion; Section 4 verifies the performance of UWB BS deployment schemes under different NLOS occlusion scenarios through simulation experiments, and the optimal arrangement scheme of underground garage under different BS numbers is introduced; and Section 5 concludes and offers an outlook.

## 2. Methods

The PSO algorithm is one of the swarm intelligence optimization algorithms inspired by the simulation of bird aggregation and fish swarming behavior, and it is a stochastic optimization method based on population searches to finding optimal solutions to single/multi-objective problems [21,22].

Compared with other intelligent optimization algorithms (genetic algorithm, artificial bee colony algorithm, etc.), the PSO algorithm has the advantages of simplicity, robustness, fast convergence, and only having a few adjustable parameters. It only needs to establish a suitable fitness function for the problem and approach the optimal solution of the problem through continuous evolution operations. The standard PSO algorithm suffers from premature convergence and poor diversity [20], and this paper provides an improved PSO algorithm that introduces a Lévy flight mutation strategy to increase the diversity of the population and prevent premature convergence.

### 2.1. Standard PSO Algorithm

In the standard PSO algorithm [22], each particle is considered as an independent solution to a massless and volumeless single-objective problem in the D-dimensional search space with its own position and velocity. First, the population particles are randomly initialized according to their position and velocity in the search space. And then, using the particle fitness function, each particle is continuously aggregated toward the optimal location in accordance with its personal historical best optimal position (pbest) and the optimal position of the entire population (gbest). The PSO evolution formula is as follows:

(1) $$\begin{array}{l} v_{i}^{d}(t+1)=\omega *v_{i}^{d}(t)+c_{1}*r_{1}*[\mathrm{pbes}t_{i}^{d}(t)-x_{i}^{d}(t)]+c_{2}*r_{2}*[\mathrm{gbes}t_{i}^{d}(t)-x_{i}^{d}(t)]\\ x_{i}^{d}(t+1)=x_{i}^{d}(t)+v_{i}^{d}(t+1) \end{array}$$

where d=1,2,…,D is the dimension of the parameter to be optimized, and the position and velocity of the i-th particle in population (i=1,2,…,N) are, respectively, $x_{i}^{d}(t)$ and $v_{i}^{d}(t)$; $pbest_{i}=(pbest_{i}^{1},pbest_{i}^{2},\cdots ,pbest_{i}^{D})$ is the i-th particle’s own best historical position; $gbest=(gbest^{1},gbest^{2},\cdots ,gbest^{D})$ is the best historical position of the entire population; ω is an inertia weight parameter used for achieving a balance between global exploration and local exploitation, decreasing linearly from large to small; $c_{1}$ and $c_{2}$ is the acceleration factor; $r_{1}$ and $r_{2}$ are two random numbers generated in the range [0,1]. That is, the particle velocity update consists of three parts. The first part is the inertia of particle maintaining its previous velocity, which can be called the “memory” part. The second part is the “cognition” part, which represents the memory of the particle of its own historical experience. The third part reflects the information sharing and cooperation between particles, which is called the “social” part.

### 2.2. Levi’s Flight Strategy

Lévy distribution is a kind of probability distribution proposed by French mathematician Lévy in the 1930s. It has been studied extensively by many scholars since then. So far, it has been proved that the foraging trajectories of many animals and insects in nature (such as albatrosses, bees, and fruit flies) conform to the Lévy distribution model [23]. Lévy flight is a random search path obeying a Lévy distribution, which is a kind of walk between a short distance search and occasionally longer distance walk, and it can explain many random phenomena in nature, such as Brownian motion, random walk, etc. Lévy flight behavior has now been used in the optimization field; for example, the cuckoo algorithm uses Lévy flight for position updates [24]. Lévy flight can expand the search space and increase population diversity, which increases the likelihood that intelligent optimization algorithms skip out of the local optimum [25]. The position update formula for Lévy flight can be expressed as follows:

(2) $$x_{i}^{\prime }(t)=x_{i}(t)+\alpha ⊕Levy(\lambda )$$

where $x_{i}(t)$ denotes the i-th solution of the t-th generation; ⊕ denotes point-to-point multiplication; α denotes the step size control factor, where $\alpha =0.01(x_{i}(t)-x_{b})$, $x_{b}$ is the current optimal solution; Levy(λ) denotes a path that obeys the Lévy distribution and meets the following formula:

(3) $$Levy(\lambda )\sim u=t^{-\lambda }\;1<\lambda \leq 3$$

where t represents the step size of each random walk, u is a random variable subject to the Levy distribution, and λ is a shape parameter of the Levy distribution that controls the distribution characteristics of the step size.

Due to the complexity of the Lévy distribution, the Mantegna algorithm is now often used to simulate it, and the Mantegna algorithm’s step size is calculated as follows:

(4) $$s=\frac{\mu }{{\left|\nu \right|}^{1/\gamma }}$$

where γ is usually the constant 1.5, and μ and ν are normally distributed. They are defined as follows:

(5) $$\begin{array}{l} \mu \sim N(0,\sigma_{\mu }^{2}),\sigma_{\mu }={\left\{\frac{\Gamma (1+\gamma )\sin(\pi \gamma /2)}{\gamma \cdot \Gamma \left[\left(\gamma +1\right)/2\right]\cdot 2^{\left(\gamma -1\right)/2}}\right\}}^{1/\gamma }\\ \nu \sim N(0,\sigma_{\nu }^{2}),\sigma_{\nu }=1 \end{array}$$

**Figure 1:**
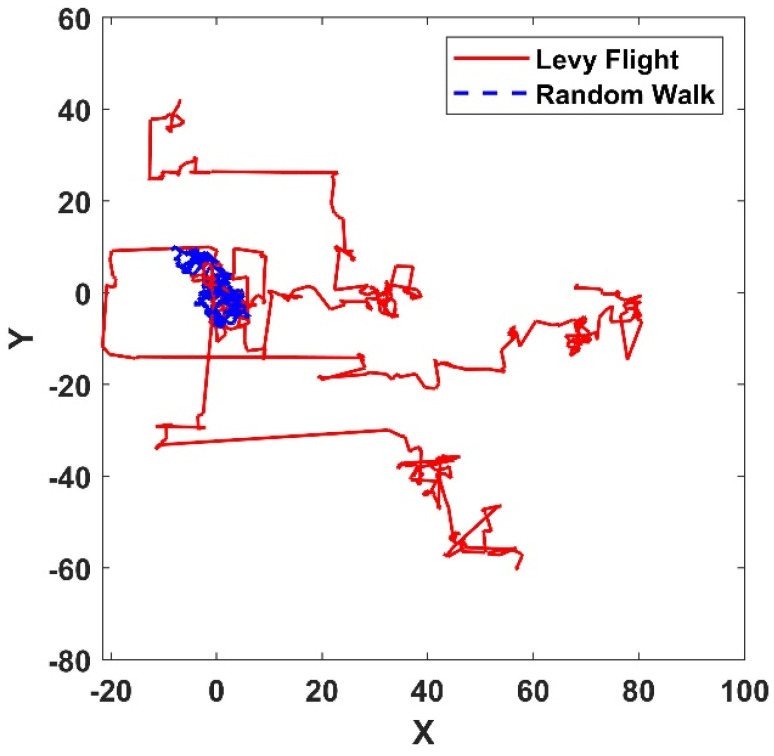
Compare Lévy flight and random walk operator results.

A large number of studies have shown that Lévy flight can improve the efficiency of searches in uncertain environments. To illustrate the superiority of Lévy flight, the difference between the Lévy flight strategy and the random walk is compared in the MATLAB R2021a software. As shown in Figure 1, Lévy flight and random walk set the step size parameter to 200, which can prove that Lévy flight has a larger search range and can be applied to the PSO algorithm to improve the dynamism and jumping ability of the particle swarm, thus improving the global optimization capability of the PSO algorithm.

### 2.3. LPSO Algorithm

To prevent the PSO from falling into local optimum values and premature convergence, the Lévy flight strategy is introduced in the standard PSO algorithm, the basic idea behind which is that it updates its own historical best position by setting and recording particle stagnation time parameter *n*. If the parameter *n* is greater than the threshold value 10, then that particle selects Equation (3) for Lévy flight to obtain a new position $x_{i}^{\prime }(t)$ and uses the greedy mechanism to compare the fitness of the old and new positions in order to retain the better solution, i.e.,

(6) $$x_{i}(t)=\left\{\begin{array}{c} x_{i}(t),fit(x_{i}(t))<fit(x_{i}^{\prime }(t))\\ x_{i}^{\prime }(t),fit(x_{i}^{\prime }(t))<fit(x_{i}(t)) \end{array}\right.$$

**Figure 2:**
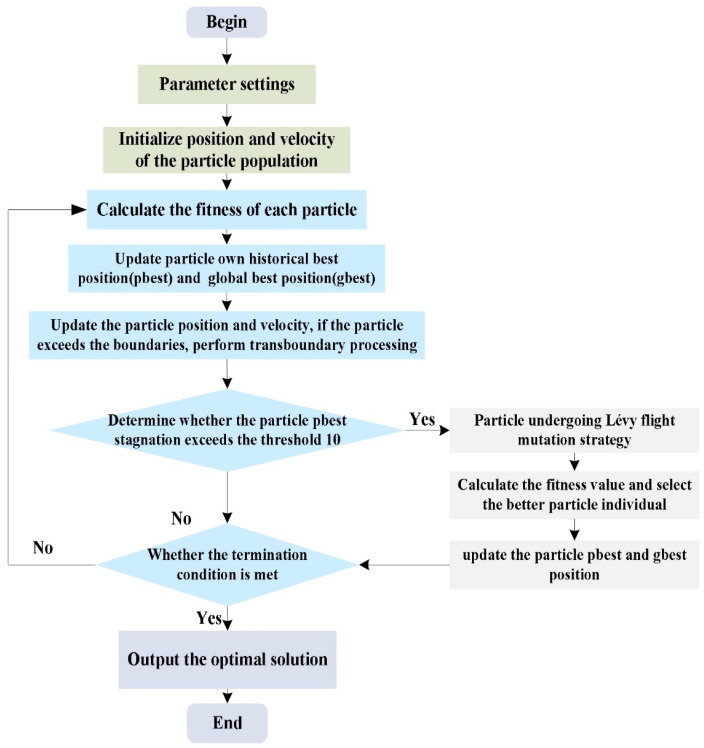
LPSO algorithm flowchart.

Figure 2 illustrates the specific procedure of the LPSO algorithm, and the specific steps are as follows:

(a) Population initialization, which initializes the position and velocity of population particles in a randomly generated manner.
(b) Calculate all particles fitness value and update the particle’s own historical best fitness value, pbest, and global best position of the whole particles, gbest.
(c) Update the velocity and position of the particle according to Equation (1), and conduct transboundary processing.
(d) Determine whether the particle pbest stagnation number n exceeds the threshold value 10; if so, then use Equation (3) to enter the Lévy flight mutation strategy to update the particle position, select the better particle individual, and update the particle’s pbest and gbest positions.
(e) Determine whether the LPSO algorithm meets the evolutionary end condition. If yes, the optimal solution is output and the algorithm search stops; if not, return to step (b) and continue the search or evolution process.

## 3. Fitness Function Model for UWB BS Deployment Optimization Considering NLOS Occlusion

The UWB BS deployment optimization problem can be modeled into a single-objective optimization problem and then solved using the LPSO algorithm outlined in Part 2. The key lies in establishing a suitable fitness function model, and this section proposes a new model for UWB BS deployment optimization, taking into account NLOS occlusion. At present, most indoor positioning demands are focused on the plane direction, and the demands in the height direction are smaller. Therefore, this paper only considers the UWB BS and tag deployment in the planes of approximately equal height and optimizes the best deployment location of the UWB BS.

### 3.1. UWB PDOP Calculation Principle

**Figure 3:**
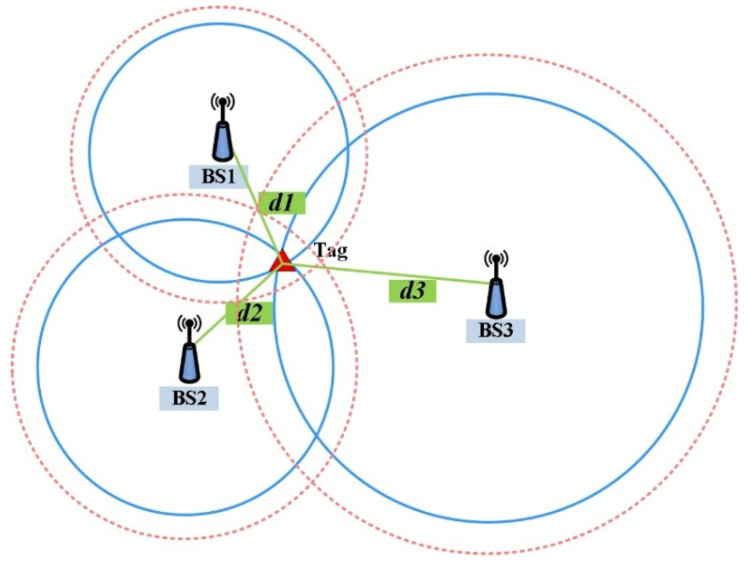
UWB measurement range value positioning principle, the blue solid line represents the true value of the measurement range, ideally the three circles meet at a point, and the pink dashed line represents the actual measurement range value containing the error, and the three circles meet to form a region.

UWB positioning systems are widely used to obtain mobile tag positions using Time-of-Arrival (TOA)/Time-of-Flight (TOF)/two-way-ranging (TWR) measurements. As shown in Figure 3, the basic UWB positioning principle using TOF in the two-dimensional plane is the intersection of multiple circles to calculate tag position coordinates.

Set $BS_{i}(X_{i},Y_{i}),i=1,2,\cdots ,N$ as the location of the UWB BS. The tag’s location is (x,y) and its measurement distance to the BS is $d_{i}$, respectively. At least three UWB BSs are needed to establish the following observation equations:

(7) $$\left\{\begin{array}{c} d_{1}=\sqrt{{\left(X_{1}-x\right)}^{2}+{\left(Y_{1}-y\right)}^{2}}+\varepsilon_{1}\\ d_{2}=\sqrt{{\left(X_{2}-x\right)}^{2}+{\left(Y_{2}-y\right)}^{2}}+\varepsilon_{2}\\ \vdots \\ d_{i}=\sqrt{{\left(X_{i}-x\right)}^{2}+{\left(Y_{i}-y\right)}^{2}}+\varepsilon_{i} \end{array}\right.$$

The approximate position of tag $\left(x_{0},y_{0}\right)$ can be obtained using the Caffery’s Linear Localization with Optimization algorithm (Caffery-LLOP) [26], and Equation (8) is linearly expanded by the Taylor series, retaining the first-order terms to obtain the following:

(8) AX=l,P

where $A=\left[\begin{array}{ll} \frac{X_{1}-x_{0}}{d_{1}^{0}} & \frac{Y_{1}-y_{0}}{d_{1}^{0}}\\ \frac{X_{2}-x_{0}}{d_{2}^{0}} & \frac{Y_{2}-y_{0}}{d_{2}^{0}}\\ \vdots & \vdots \\ \frac{X_{i}-x_{0}}{d_{i}^{0}} & \frac{Y_{i}-y_{0}}{d_{i}^{0}} \end{array}\right]$, $X=\left[\begin{array}{c} dx\\ dy \end{array}\right]$, and $l=\left[\begin{array}{c} d_{1}-d_{1}^{0}\\ d_{2}-d_{2}^{0}\\ \vdots \\ d_{i}-d_{i}^{0} \end{array}\right]$. The weighted least squares algorithm in Equation (9) is adopted to iterate calculations until the threshold value is satisfied.

(9) $$X={\left(A^{T}PA\right)}^{-1}A^{T}Pl$$

(10) $$HDOP=\sqrt{tr{\left(A^{T}A\right)}^{-1}}$$

The UWB positioning error is affected by the geometric deployment of the BS in addition to the ranging error. Equation (10) gives the formula for calculating the horizontal dilution of precision (HDOP), which is essentially the amplification effect of the final positioning error on the ranging error, namely:

(11) $$HDOP=\frac{\sqrt{\sigma_{x}^{2}+\sigma_{y}^{2}}}{\sigma }$$

where $\sigma_{x}^{2}$ and $\sigma_{y}^{2}$ represent the error covariance in the x and y directions, respectively; σ stands for the standard deviation of the measured distance. Most studies have traditionally focused on the deployment of UWB BSs in line-of-sight (LOS) environments, but in real-world applications, such as large equipment obstructions in factories, NLOS scenarios are more common. The NLOS signal seriously affects the UWB positioning accuracy and the coverage of the locatable space, and there are few UWB BS deployment schemes that take NLOS into account.

### 3.2. Locatable Signal Coverage Rate Calculation Model

In UWB 2D indoor positioning, the locatable space coverage rate of UWB signals is a significant indicator. The mobile tag must receive at least three LOS signals to be considered a valid positioning point; otherwise, it is considered a blind positioning point. In this paper, a planar grid segmentation method is used to segment the indoor positioning area, and the indoor spatial positioning signal coverage is calculated using the K-coverage model [27,28].

Suppose the area of the indoor positioning plane is S, the length is L, the width is W, and the length of the small grid side is l, and that the indoor plane area S is divided into M∗N small grids, namely, M=L/l and N=W/l. Let the central coordinate of each grid be $C_{m,n}(x_{m},y_{n})$, m=(1,⋯,M),n=(1,⋯,N). The grid area is replaced by the coordinates of this center point, that is, the coverage rate of each small grid area is calculated using center point $C_{m,n}(x_{m},y_{n})$ approximation. Set $BS=(BS_{1},BS_{2},\cdots ,BS_{N})$ as the location set of UWB BS. The UWB BS ranging coverage radius is R and $d_{i}=\sqrt{{\left(X_{i}-x_{m}\right)}^{2}+{\left(Y_{i}-y_{n}\right)}^{2}}$ is the distance between $BS_{i}(X_{i},Y_{i})$ and grid point $C_{m,n}(x_{m},y_{n})$ if $d_{i}\leq R$ indicates that grid point $C_{m,n}$ is covered by $BS_{i}$. And when the effective coverage of BS for this grid point is greater than 3, the grid point can be located, and the ratio of all the locatable grid points is calculated as the whole space coverage rate.

### 3.3. Optimal Fitness Function Model for UWB BS Deployment

In order to avoid or minimize the influence of NLOS on UWB tag positioning accuracy, this paper proposes a new UWB BS deployment optimization model, i.e., the locatable spatial coverage rate in the whole indoor space is used as the fitness function, and the final optimal BS deployment location is obtained by the LPSO algorithm in Section 2.3. The following is an example of a rectangular indoor factory environment used to construct the fitness function, considering NLOS occlusion, with the following steps.

**Figure 4:**
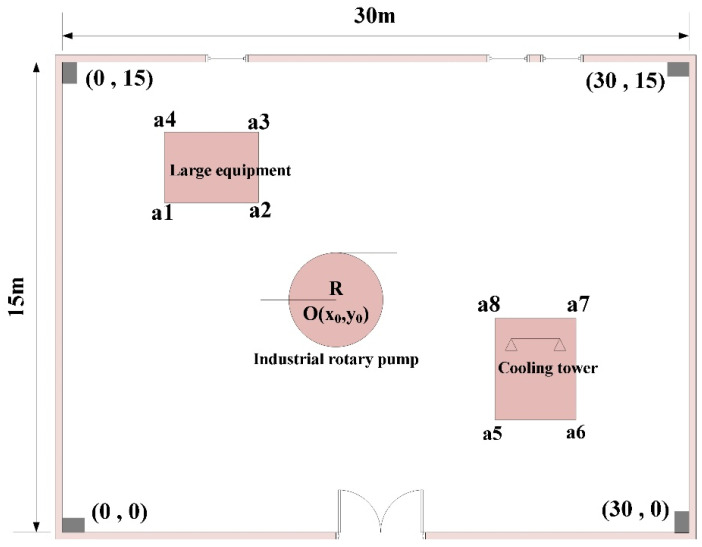
Indoor space plane map and shade plane projection.

Step 1: The plane map of the indoor area of a factory is shown in Figure 4, along with the occlusions that are present. This indoor space contains fixed facilities such as large equipment, industrial rotary pumps, and cooling towers, which are high and impenetrable to the signal, with rectangular and circular plan projections. The coordinates of the vertices of the large equipment are a1, a2, a3, and a4, the center and radius of the industrial rotary pump are O and R, and the coordinates of the vertices of the cooling tower are a5, a6, a7, and a8.

**Figure 5:**
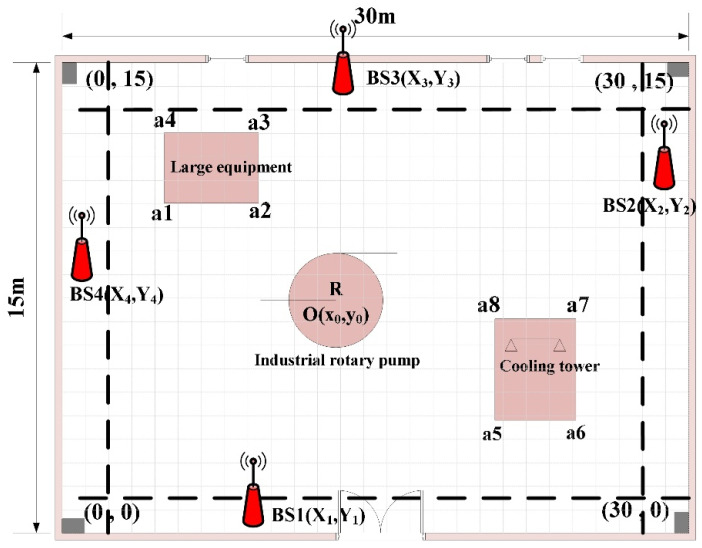
UWB BS deployable range and indoor positioning space gridding.

Step 2: Due to the features of indoor scenes, UWB BSs need to be deployed in the area near the wall in general to meet the requirements of power supply and wiring. Assuming a total of 4 BSs are deployed, BS coordinates $BS_{i}(X_{i},Y_{i})(i=1,2,3,4)$ are randomly generated according to the deployment range of BS and the indoor space to be located is discretely divided according to the grid length of l×l, resulting in a total of M×N grids. The gridding of the area to be located and the deployment range of the regional BSs are given in Figure 5.

**Figure 6:**
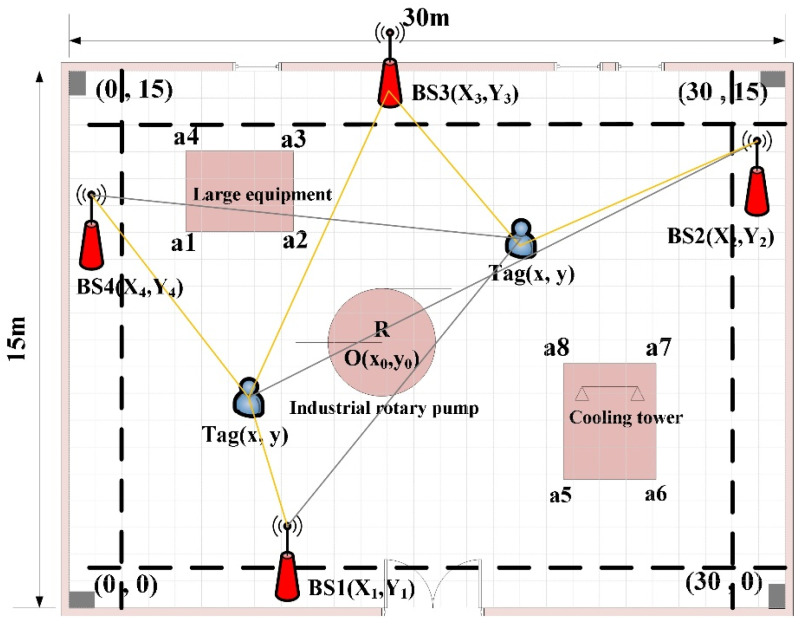
Schematic diagram of grid point coverage availability determination under NLOS occlusion. The yellow solid line ranging signal is not blocked, the Tag can be seen between the UWB base station, and the signal can be direct; The gray solid line indicates that there is no visibility between the Tag and the UWB base station, and the measurement ranging signal cannot be directly affected by the occlusion.

Step 3: Iterate the grid center point location $Tag(x_{m},y_{n}),1\leq m\leq M,1\leq n\leq N$ in turn, as shown in Figure 6. Calculate the distance $d_{i}=\sqrt{{\left(X_{i}-x_{m}\right)}^{2}+{\left(Y_{i}-y_{n}\right)}^{2}},\;i=1,2,3,4$ from this grid point to all BSs, determine whether $d_{i}$ is less than the effective coverage distance R of the UWB BS, and record the visible state of the BS, namely:

(12) $$P(i)=\left\{\begin{array}{cc} 1 & ,d_{i}\leq R\\ 0 & ,d_{i}>R \end{array}\right.$$

Step 4: Determine the NLOS situation of the visual BS in step (3), and judge the intersection of the line segment between each visual BS and $Tag(x_{m},y_{n})$ and the line segment between the peripheral vertices of each occlusion area. The grid point is only visible to the BS (LOS) when they are not intersecting (i.e, L(i)=1), as shown in Equation (13). Otherwise, the BS is marked as NLOS BS (i.e, L(i)=0). In this way, all BSs and all occluded areas are marked cyclically.

(13) $$L(i)=\left\{\begin{array}{cc} 1 & ,LOS\\ 0 & ,NLOS \end{array}\right.$$

Step 5: Combine the number of all visible BSs in step (3) and deduct the NLOS BSs in step (4) to indicate the final visible BSs set E=P∩L. In 2D positioning, the grid point with TOA/TOF/TWR as the ranging observation requires the final number of visible BSs to be at least 3 to be locatable, and the HDOP value of the point is calculated. At the same time, considering the positioning requirements of the indoor space (e.g., the ranging error is fixed at 10 cm, and the limitation of positioning accuracy is better than 30 cm, so the HDOP value is limited to less than 3), the coverage status of the grid point is marked, namely:

(14) $$HDOP(m,n)=\left\{\begin{array}{cc} \sqrt{tr{\left(A^{T}A\right)}^{-1}} & ,E\geq 3\\ 0 & ,E<3 \end{array}\right.$$

(15) $$cov(m,n)=\left\{\begin{array}{l} 1\;,E\geq 3\cap HDOP(m,n)\leq \delta \\ 0\;,E<3 \end{array}\right.$$

where δ=3 denotes HDOP value threshold of the positioning demand.

Step 6: The LPSO fitness function is constructed, i.e., the coverage rate of all locatable grid points is calculated using Equation (16). The larger coverage rate indicates the smaller blind positioning area and larger available positioning space, which is fed into the LPSO algorithm in Section 2.3 for searching and updating until the optimization meets the termination condition, and outputs the best combination of BS deployment locations.

(16) $$fitness=\frac{\sum_{P\in M\times N}cov(m,n)}{M\times N}$$

Step 7: Output the UWB BS optimal deployment result in this area, evaluate whether the result meets the requirements, and adjust the scheme according to the actual building deployment and characteristics in the scene. If it does not meet the requirements, repeat the above steps (2~6) until the requirements are met.

## 4. Result and Discussion

### 4.1. Experiment 1: Performance Verification Test

#### 4.1.1. Simulation Experimental Data and Parameter Settings

As shown in Figure 4, Figure 5 and Figure 6, the rectangular indoor scene size in the simulation factory is 30 m × 15 m, with the origin in the lower left corner (0 m,0 m). The coordinates of the other three vertices are (30 m,0 m), (30 m,15 m), and (0 m,15 m), respectively, when seeking to set the length of the grid to l=0.2 m, and all BSs can be deployed in the range of 1 m near the wall. Based on the number of occlusions, four different occlusion schemes were set up in the experiment (i.e., NLOS = 0, NLOS = 1, NLOS = 3 and NLOS = 5). The plane coordinates corresponding to various occlusions were obtained in advance via the map. In this paper, the outcomes of rectangular deployment patterns, diamond deployment patterns, standard PSO deployment, and LPSO deployment are compared and analyzed. The parameter settings of the standard PSO algorithm and LPSO algorithm are consistent, with a population size of 20 and an iteration or evolution time of 100.

#### 4.1.2. Experimental Results and Analysis

**Figure 7:**
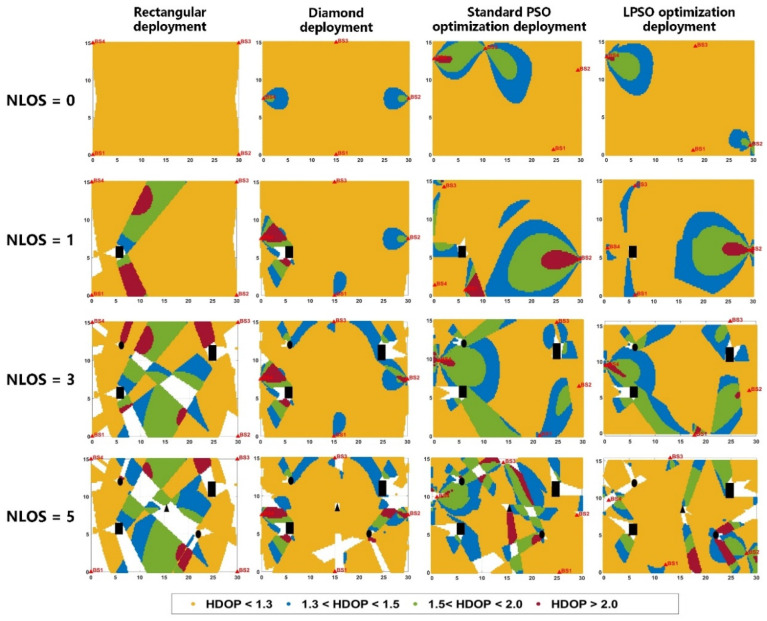
Comparison of HDOP values of four different UWB BS layout models under different NLOS conditions, where black rectangles, triangle and circles represent impenetrable obstacles and white areas represent blind areas that cannot be located.

The experiments sequentially emulated four distinct scenarios: an indoor open space without any obstructions (NLOS = 0), an indoor space with a single non-penetrable device or column (as depicted in the black rectangular area in Figure 7) (NLOS = 1), an indoor space with three non-penetrable obstruction areas (black rectangles and circles in Figure 7) (NLOS = 3), and an indoor space with five non-penetrable obstruction areas (black rectangles, circles, and triangles in Figure 7) (NLOS = 5). Figure 7 illustrates the spatial distribution of HDOP values across the four layout configurations. Red triangles denote the locations of the BS, while blank areas indicate blind zones where positioning is not feasible. The HDOP values at the center points of various spatial grids are represented by yellow, blue, green, and bright red circles. Table 1 presents the coverage rate and average HDOP values for the four UWB BS layout schemes under the different NLOS conditions.

As depicted in Figure 7 and detailed in Table 1, under conditions with no NLOS obstruction (NLOS = 0), the rectangular deployment model exhibits a minimal localization blind area, achieving a 97.1% locatable space coverage rate. In contrast, both the diamond deployment and the PSO-optimized deployment models boast a full 100% locatable space coverage rate. The average HDOP value for the locatable area of the rectangular deployment is 1.14, while the diamond deployment model has the lowest average HDOP value at 1.09. The LPSO algorithm’s average HDOP value is 1.11, outperforming the standard PSO algorithm and ranking second. Thus, while the rectangular deployment has a specific blind positioning region, the diamond deployment and the two PSO-optimized deployments excel in an unobstructed environment. In the presence of an impenetrable occlusion within the space to be located (NLOS = 1), the locatable area coverage rate for the rectangular deployment model drops to 94.8%, and it is 97.5% for the diamond deployment. However, the optimized deployment models using both the standard PSO algorithm and the LPSO algorithm maintain coverage rates above 99.0%. Concurrently, the LPSO algorithm’s average HDOP value is 1.24, which is significantly better than that of the standard PSO algorithm.

When the number of NLOS occlusions is 3, the rectangular deployment model exhibits a significantly large blind localization area. Consequently, the coverage rate of the locatable area drops to 77.7%. In contrast, the diamond deployment model achieves a locatable space coverage rate of 93.7%. Both the standard PSO deployment and the LPSO deployment models demonstrate a locatable space coverage rate exceeding 96.0%. Notably, the LPSO deployment model’s locatable space coverage rate shows an improvement of 19.0% over the rectangular deployment model. Moreover, the average HDOP value of 1.21 achieved by the LPSO algorithm outperforms the standard PSO algorithm’s average HDOP value of 1.29.

When NLOS occlusions reach 5, the locatable space coverage rate of the rectangular deployment model further decreases to only 67.0%, resulting in a substantial blind area for UWB tag localization or positioning. The diamond deployment model, however, outperforms the rectangular deployment model with a coverage rate of 83.1%. The deployment model optimized by the standard PSO algorithm attains a locatable space coverage rate of 88.3%, while the highest locatable space coverage rate of 89.8% is achieved by the LPSO-optimized deployment model presented in this paper. Meanwhile, the average HDOP values of the four deployment schemes are approximately equal to 1.3, a value which meets the requirements for positioning. In more severe occlusion environments, traditional rectangular and diamond deployments have larger blind positioning areas. In contrast, the deployment optimization model proposed in this paper, when solved by the standard PSO and LPSO algorithms while considering NLOS occlusions, can obtain a higher level of coverage. The two deployment schemes are more flexible and efficient, and are undoubtedly superior to the traditional deployment models.

**Figure 8:**
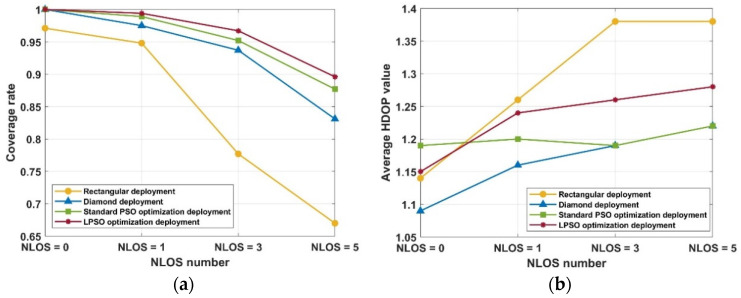
The locatable space coverage rate (**a**) and average HDOP value (**b**) variation curves of four UWB BS deployment schemes under different NLOS occlusion scenarios.

Figure 8 gives the locatable space coverage rate and average HDOP value variation curves of the four UWB BS deployment schemes under different NLOS occlusion scenarios. From a comprehensive view, as the NLOS occlusions gradually increase, the coverage rate of all four deployment models gradually decreases and the location blindness gradually increases, and the rectangular and diamond deployment models exhibit a more pronounced coverage reduction than the two PSO deployment models. The average HDOP value gradually becomes bigger as the NLOS occlusions increase, but the average HDOP value of the four schemes is great than 1.5, which meets the demands of UWB tag high-precision positioning. The above results show that the rectangle deployment has the worst coverage, followed by the diamond deployment, and that the rectangle and diamond deployment are no longer advantageous in the complex environment with severe occlusion. The new deployment optimization model of UWB BS proposed in this paper, which takes into account the influence of NLOS, achieves the highest locatable space coverage rate in the environment with or without NLOS occlusion, greatly reduces the location blind area, and has a small average HDOP value, which illustrates the superiority of the model in this paper.

**Figure 9:**
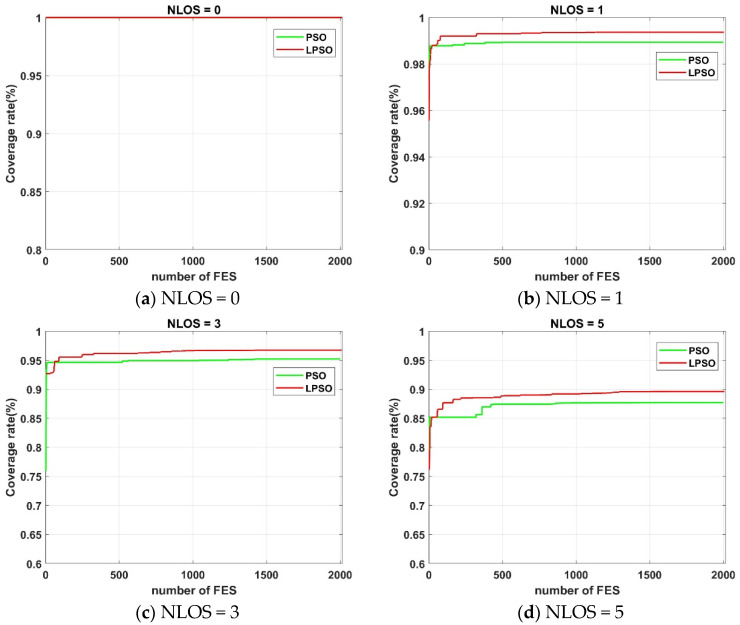
The locatable space coverage rate of the standard PSO-optimized layout model and the LPSO-optimized layout model under four different NLOS occlusion conditions.

Figure 9 illustrates the variation curve of the locatable space coverage rate for both the standard PSO deployment model and the LPSO deployment model under four distinct NLOS occlusion conditions. It can be observed that the LPSO deployment model exhibits faster convergence compared to the standard PSO model across different NLOS occlusion scenarios. As the number of NLOS occlusions increases, the performance gap between the two models widens progressively. This clearly demonstrates that the proposed improved PSO algorithm, which is based on Lévy Flight optimization, possesses superior population diversity and is capable of exploring a more extensive search space, thereby achieving better convergence performance.

### 4.2. Experiment 2: Deployment Experiment of UWB BS in Underground Garage

**Figure 10:**
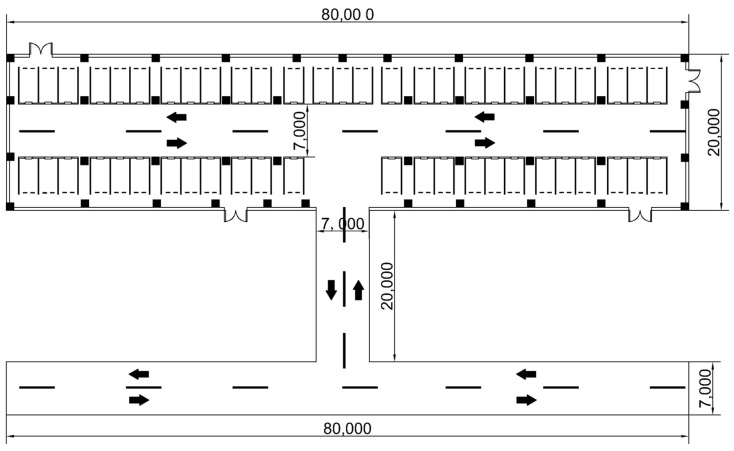
A plane diagram of an example of an underground parking lot, the arrows in the figure indicate the direction of the vehicle.

In order to verify the generalization of the UWB BS layout strategy proposed in this paper in a complex environment, the overall UWB BS layout scheme proposed in this paper was extended to a large underground garage area with an irregular shape. The plane diagram of the underground garage area is shown in Figure 10. The garage can be disassembled into multiple sub-areas such as parking lot entrances, entrance and exit ramps, and indoor areas, which can be understood as a combination of various rule modules when the BS is arranged. According to the positioning requirements of different regions, this paper will provide a comprehensive performance comparison of different UWB BS layout schemes under different numbers of BSs.

#### 4.2.1. Parameter Setting and Layout Strategy

The underground garage area scene takes the lower left corner as the origin (0 m, 0 m), and the coordinates of the other three vertices are (80 m, 0 m), (80 m, 47 m), and (0 m, 47 m), respectively. The grid length l is set to be 0.5 m, and the effective coverage radius R of the UWB BS in the case of visual distance is set to be 30 m. The layout height of the BS is 2.6 m, the tag placement height is about 2 m, the population size of the LPSO algorithm is 20, and the number of iterations is set to 100. Other parameters are the same as those set in the preceding section.

**Figure 11:**
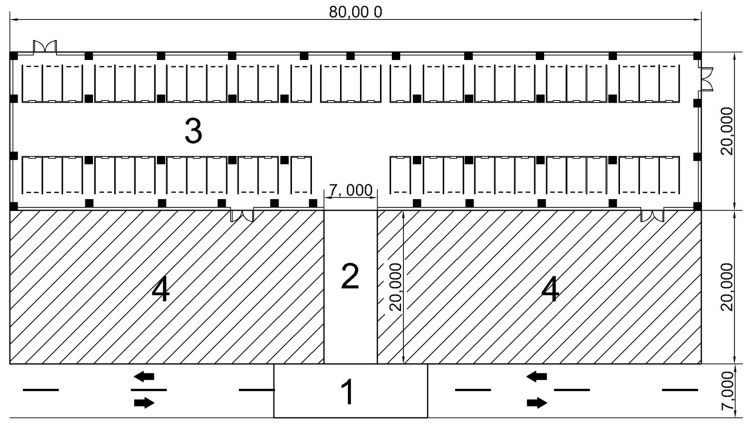
Underground parking area division, the arrows in the figure indicate the direction of the vehicle.

In this specific example, based on the unique environmental characteristics and station layout requirements of each sub-area of the parking lot, all the areas to be located are divided into four sub-areas, as shown in Figure 11. In order to ensure that each sub-area can meet its positioning requirements, targeted BS layout strategies and schemes should be proposed based on the actual situation. The BS layout methods of the four sub-areas are as follows:

(1) Area 1 is the entrance and exit area of the parking lot, with a length of 20 m and a width of 7 m. It is the connection node between the urban road and the underground garage, and the vehicle driving environment is relatively complex. Based on the LPSO algorithm, the preliminary solution was used to establish that the BS layout method approximates the rectangular distribution. Considering the environmental characteristics of the large daily average vehicle flow in this region combined with the actual scene, in order to reduce the impact of the flow of personnel and vehicles and to make the wiring beautiful, a BS distribution scheme was adopted on both sides of the road, and a total of 2 pairs of 4 BSs were laid.
(2) Area 2 is the entrance and exit ramp of the parking lot, with a length of 7 m and a width of 20 m. It is an area where vehicles frequently enter and exit the parking lot. At the same time, vehicles travel at a fast speed and have high positioning accuracy requirements. Similar to area 1, in order to ensure the coverage and accuracy of the BSs in this area, the HDOP value is the lowest when the BSs are uniformly placed. At the same time, considering the safety of driving and positioning equipment, the BSs are distributed in rectangles on both sides of the ramp inlet and outlet, with a total of 4.
(3) Area 3 is the indoor area of the parking lot, with a relatively closed space and a complex structure. In this area, the driver needs to quickly find the parking position of the vehicle through the navigation and positioning function, which puts forward high requirements for the layout model of the UWB BS. The whole area is 80 m long and 20 m wide, which can be understood as a large rectangular area. Therefore, the LPSO algorithm proposed in this paper is adopted to optimize the BS location.
(4) Area 4 is the blocked area of the concrete wall that is seriously disturbed and cannot be penetrated by the UWB BS. As such, the cocoa is set to contain two impenetrable blocks (NLOS = 2), both 35 m in length and 20 m in width.

Considering that ramp entrances and exits are the key nodes connecting area 1 and area 3, in order to connect positioning devices closely and reduce the number of BSs as much as possible, the location of BSs is coordinated and shared, and several BSs with similar locations are merged. After comprehensive adjustment, the total number of artificial BSs in area 1 and area 2 is 6. The coordinates are BS1 (36.5 m, 7 m), BS2 (43.5 m, 7 m), BS3 (30 m, 0 m), BS4 (50 m, 0 m), BS5 (36.5 m, 27 m), and BS6 (43.5 m, 27 m), wherein BS1 and BS2 are the common BSs for area 1 and area 2. BS3 and BS4 are the common BSs for area 2 and area 3.

#### 4.2.2. Evaluation of Layout Optimization Effect Under Different Number of BSs

**Figure 12:**
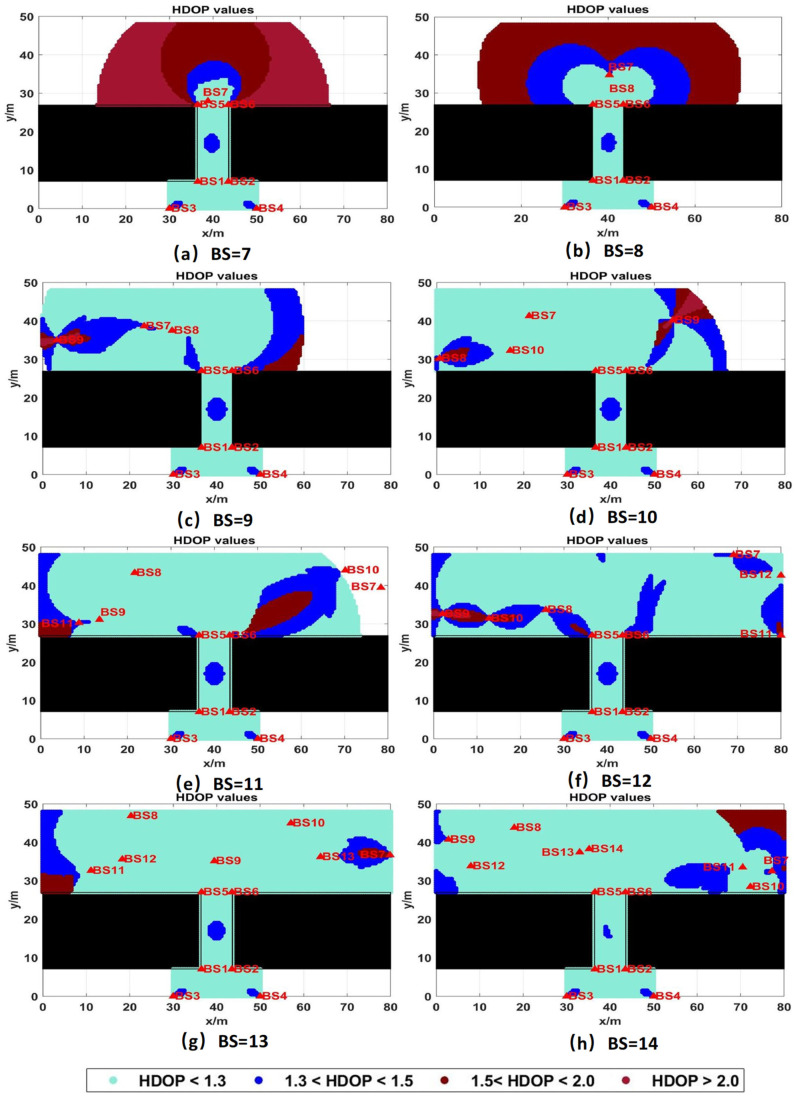
Comparison of HDOP values of underground garage layout under different numbers of BSs, where black rectangles, triangle and circles represent impenetrable obstacles and white areas represent blind areas that cannot be located.

During the process of tag positioning, the number of BSs significantly impacts positioning accuracy. When the number of BSs is limited, although the cost of BS deployment can be reduced, there is a risk of compromised accuracy and potential positioning failures. Conversely, deploying a large number of BSs can enhance positioning accuracy and coverage rate, but will lead to a substantial increase in the cost of positioning infrastructure and may result in resource waste. Therefore, when planning the BS layout, it is crucial to balance various factors to determine the optimal number of BSs. As discussed in the previous section, a total of 6 BSs were deployed in area 1 and area 2, with the total number of available BSs ranging from 7 to 14. The LPSO algorithm is employed to optimize the layout of BSs, calculating the optimal locations and fitness values for different numbers of BS deployments. Figure 12 illustrates the HDOP values of the underground garage layout under varying numbers of BSs, while Table 2 presents the comprehensive performance of the LPSO-optimized BS layout scheme.

**Figure 13:**
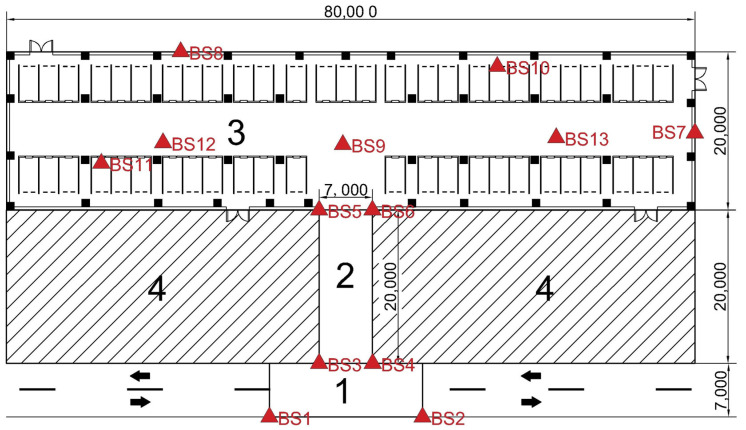
The optimal locations of the UWB BSs in scheme 7 (BS = 13) in the underground garage.

As shown in Figure 12 and Table 2, in a large underground garage with NLOS in mind, when the number of UWB BSs is 12 or more, the coverage rate is 100%. Therefore, in order to avoid information redundancy and waste in the positioning of multiple BSs and improve the positioning accuracy of UWB BSs as much as possible, scheme 7 (BS = 13) is selected as the optimal layout scheme in this case. As can be seen from Table 2, compared with scheme 1, which has the highest fitness function value and the lowest number of BSs, the optimal scheme 7 is selected, the coverage rate increases from 65.1% to 100%, and the average HDOP value decreases from 5.961 to 1.088. Scheme 7 (BS = 13) has the best comprehensive performance. The optimal location of UWB BSs are shown in Figure 13 and Table 3. These can effectively avoid the waste caused by BS redundancy and significantly improve the layout coverage and positioning accuracy of the area, which proves the practicability of the model presented in this paper.

As illustrated in Figure 12 and Table 2, in a large underground garage with NLOS conditions, when the number of UWB BSs reaches 12 or more, the coverage rate achieves 100%. To prevent redundancy and waste associated with excessive BSs while maximizing the positioning accuracy of UWB BSs, scheme 7 (BS = 13) is chosen as the optimal layout scheme in this scenario. As shown in Table 2, compared to scheme 1, which has the highest fitness function value and the fewest BSs, the optimal scheme 7 increases the coverage rate from 65.1% to 100% and reduces the average HDOP value from 5.961 to 1.088. The optimal locations of the UWB BSs in scheme 7 (BS = 13) are depicted in Figure 13 and Table 3, effectively avoiding waste caused by BS redundancy and significantly enhancing the layout coverage and positioning accuracy of the area. This demonstrates the practicality of the model presented in this paper.

## 5. Conclusions

This paper proposes a UWB BS layout optimization model that takes NLOS into account for large indoor complex scenes. Differing from the traditional classical layout model, this model constructs the overall coverage of the positioning area space as a fitness function, and adopts an improved PSO algorithm based on Levy flight to optimize the solution. The following useful conclusions can be drawn:

(1) The performance verification results show that the locatable space coverage rate of this new method is comparable to that of the diamond deployment and better than that of the rectangular deployment when there is no shading. The locatable space coverage rate of the LPSO-optimized deployment is improved by 19.0% and 22.6% compared to the rectangular deployment, and 3.0% and 6.5% compared to the diamond deployment, when the NLOS values are 3 and 5 for complex occlusion environments, respectively. Meanwhile, the LPSO deployment is better than the standard PSO deployment results and effectively decreases the blind positioning areas, while its average HDOP value is satisfactory.
(2) The deployment experiment of UWB BSs in underground garage shows that compared with the 7-BS layout method with the fewest BSs, the coverage rate of the optimal 13-BS scheme is increased by 34.9%, and the HDOP value is decreased by 81.7%, which significantly improves the regional coverage rate. The comprehensive performance of the UWB layout station is greatly improved by fully taking into account the impact of the number of BSs and NLOS shielding.

In summary, the new UWB BS layout optimization model proposed in this paper, taking into account the influence of NLOS, has important application value for realizing the flexible layout of UWB BSs in complex environments (factories, underground garages, et al.), which can effectively improve the positioning accuracy and area coverage, and reduce the influence of NLOS occlusion on positioning. Subsequently, we will introduce multiple evaluation indicators to promote the overall UWB layout scheme as a multi-objective joint optimization method in a large range of complex environments and carry out real experimental verification.

## Figures and Tables

**Table 1:** Coverage rate and average HDOP values of four different UWB BS layout schemes under different NLOS conditions.

Parameter	Occlusion Condition	Rectangular Deployment	Diamond Deployment	Standard PSO Deployment	LPSO Deployment
Coverage Rate	NLOS = 0	97.1%	100%	100%	100%
	NLOS = 1	94.8%	97.5%	99.0%	99.4%
	NLOS = 3	77.7%	93.7%	96.4%	96.7%
	NLOS = 5	67.0%	83.1%	88.3%	89.8%
Average HDOP	NLOS = 0	1.14	1.09	1.17	1.11
	NLOS = 1	1.26	1.16	1.34	1.24
	NLOS = 3	1.38	1.19	1.29	1.21
	NLOS = 5	1.38	1.22	1.29	1.24

**Table 2:** Comparison of comprehensive performance of UWB BS layout scheme under different numbers of BSs.

Scheme Index	1	2	3	4	5	6	7	8
Number of BSs	7	8	9	10	11	12	13	14
Coverage Rate	65.1%	76.2%	76.9%	82.5%	90.0%	100%	100%	100%
Average HDOP value	5.961	2.347	1.365	1.258	1.305	1.265	1.088	1.146
Fitness value	1.536	1.313	1.299	1.216	1.112	1.000	1.000	1.000

**Table 3:** UWB BS coordinate values in scheme 7 in the underground garage.

BS	Coordinate Value/m	BS	Coordinate Value/m
BS1	(36.5, 7)	BS8	(20.3, 46.8)
BS2	(43.5, 7)	BS9	(39.4, 35.1)
BS3	(30, 0)	BS10	(57.1, 45.0)
BS4	(50, 0)	BS11	(11.1, 32.6)
BS5	(36.5, 27)	BS12	(18.3, 35.6)
BS6	(43.5, 27)	BS13	(63.9, 36.2)
BS7	(79.9, 36.7)		

## Data Availability

The datasets used and analyzed during the current study are available from the corresponding author on a reasonable request.

## References

[1] Wang S.L., Dong X.S., Liu G.Y., Gao M., Xiao G.W., Zhao W.H., Lv D. (2022). GNSS RTK/UWB/DBA Fusion Positioning Method and Its Performance Evaluation. Remote Sens..

[2] Liu T., Liu J., Wang J., Zhang H., Zhang B., Ma Y., Sun M., Lv Z., Xu G. (2023). Pseudolites to Support Location Services in Smart Cities: Review and Prospects. Smart Cities.

[3] Cheng Y., Li F., Chen J.X., Ji W. A Smart Parking System using WiFi and Wireless Sensor Network. Proceedings of the 3rd IEEE International Conference on Consumer Electronics-Taiwan (ICCE-TW).

[4] Faragher R., Harle R. (2015). Location Fingerprinting with Bluetooth Low Energy Beacons. IEEE J. Sel. Areas Commun..

[5] Zhuang Y., Yang J., Li Y., Qi L.N., El-Sheimy N. (2016). Smartphone-Based Indoor Localization with Bluetooth Low Energy Beacons. Sensors.

[6] Khalili B., Abbaspour R.A., Chehreghan A., Vesali N. (2022). A Context-Aware Smartphone-Based 3D Indoor Positioning Using Pedestrian Dead Reckoning. Sensors.

[7] Piciarelli C. (2016). Visual Indoor Localization in Known Environments. IEEE Signal Process Lett..

[8] Rafian P., Legge G.E. (2017). Remote Sighted Assistants for Indoor Location Sensing of Visually Impaired Pedestrians. ACM Trans. Appl. Percept..

[9] Alarifi A., Al-Salman A., Alsaleh M., Alnafessah A., Al-Hadhrami S., Al-Ammar M.A., Al-Khalifa H.S. (2016). Ultra wideband indoor positioning technologies: Analysis and recent advances. Sensors.

[10] Bastida-Castillo A., Gómez-Carmona C.D., De La Cruz Sánchez E., Pino-Ortega J. (2019). Comparing accuracy between global positioning systems and ultra-wideband-based position tracking systems used for tactical analyses in soccer. Eur. J. Sport Sci..

[11] Khosravi F. (2019). Positioning of the Wheel Loader by Ultra Wideband Technology.

[12] Yavari M., Nickerson B.G. (2014). Ultra Wideband Wireless Positioning Systems.

[13] Jourdan D.B., Dardari D., Win M.Z. (2008). Position error bound for UWB localization in dense cluttered environments. IEEE Trans. Aerosp. Electron. Syst..

[14] Yu K., Wen K., Li Y., Zhang S., Zhang K. (2018). A Novel NLOS Mitigation Algorithm for UWB Localization in Harsh Indoor Environments. IEEE Trans. Veh. Technol..

[15] Zhang F., Li H., Ding Y., Yang S.H., Yang L. (2021). Dilution of precision for time difference of arrival with station deployment. IET Signal Proc..

[16] Chen W.J., Narayanan R.M. (2011). Antenna Placement for Minimizing Target Localization Error in UWB MIMO Noise Radar. IEEE Antennas Wirel. Propag. Lett..

[17] Levanon N. (2000). Lowest GDOP in 2-D scenarios. IEE Proc.-Radar Sonar Navig..

[18] Jiang S., Zhao C., Zhu Y., Wang C., Du Y. (2022). A Practical and Economical Ultra-wideband Base Station Placement Approach for Indoor Autonomous Driving Systems. J. Adv. Transp..

[19] Wang M., Chen Z., Zhou Z., Fu J.L., Qiu H.B. (2020). Analysis of the Applicability of Dilution of Precision in the Base Station Configuration Optimization of Ultrawideband Indoor TDOA Positioning System. IEEE Access.

[20] Yu X.M., Wang H.Q., Lv H.W., Liu X.B., Wu J.Q. (2019). A fusion optimization algorithm of network element layout for indoor positioning. EURASIP J. Wirel. Commun. Netw..

[21] Wang S.L., Liu G.Y., Gao M., Cao S.L., Guo A.Z., Wang J.C. (2020). Heterogeneous comprehensive learning and dynamic multi- swarm particle swarm optimizer with two mutation operators. Inform. Sci..

[22] Kennedy J., Mendes R. (2002). Population structure and particle swarm performance. Proceedings of the IEEE Congress on Evolutionary Computation.

[23] Yang X.S., Deb S. (2010). Engineering Optimisation by Cuckoo Search. Int. J. Math. Model. Numer. Optim..

[24] Gandomi A.H., Yang X.S., Alavi A.H. (2013). Cuckoo search algorithm: A metaheuristic approach to solve structural optimization problems. Eng. Comput..

[25] Hakli H., Uguz H. (2014). A novel particle swarm optimization algorithm with Levy flight. Appl. Soft Comput..

[26] James J., Caffery J. (2000). Wireless Location in CDMA Cellular Radio Systems.

[27] Wang Y.C., Tseng Y.C. (2008). Distributed deployment schemes for mobile wireless sensor networks to ensure multilevel coverage. IEEE Trans. Parallel Distrib. Syst..

[28] Ammari H.M., Das S.K. (2012). Centralized and Clustered k-Coverage Protocols for Wireless Sensor Networks. IEEE Trans. Comput..

